# Measuring body image during pregnancy: psychometric properties and validity of a German translation of the Body Image in Pregnancy Scale (BIPS-G)

**DOI:** 10.1186/s12884-019-2386-4

**Published:** 2019-07-12

**Authors:** Michaela Nagl, Lene Jepsen, Katja Linde, Anette Kersting

**Affiliations:** Department of Psychosomatic Medicine and Psychotherapy, Medical Faculty, University of Leipzig, Semmelweisstraße 10, 04103 Leipzig, Germany

**Keywords:** Body image, Pregnancy, Assessment, Translation, Psychometrics, Validation

## Abstract

**Background:**

This study aimed to provide a German translation of the Body Image in Pregnancy Scale (BIPS) – a measure tailored to pregnancy-specific aspects of body image while being consistent to the multifaceted theoretical framework of body image – and to test its psychometric properties and validity.

**Methods:**

The English-language original version of the BIPS was translated into German language using a forward-backward translation rationale. Face validity of the items was tested in cognitive interviews (*n* = 5). An online survey was conducted among 291 pregnant women. After conducting standard item analyses, factorial validity was tested using principal-axis factor analysis (PAF). Convergent and incremental validity with measures of body dissatisfaction (FFB), depression (EPDS), anxiety (GAD-7), self-esteem (RSE), and eating disorder psychopathology (EDE-Q) was tested by bivariate correlations and multiple linear hierarchical regression analyses.

**Results:**

The PFA revealed a 32 item and 6-factor solution resembling the dimensions preoccupation with appearance, dissatisfaction with strength-related aspects, dissatisfaction with body parts, dissatisfaction with complexion, prioritization of appearance over function, and concerns about sexual attractiveness. Internal consistency on a subscale level was good to excellent (.79 ≤ Cronbach’s α ≤ .91). Consistent with theoretical assumptions, we found significant positive correlations of BIPS-G subscales with depression, anxiety and eating disorder psychopathology and negative correlations of BIPS-G subscales with self-esteem. BIPS-G subscales substantially improved the prediction of depression, anxiety, self-esteem and eating disorder psychopathology over demographic factors and body dissatisfaction (.03 ≤ ΔR^2^ ≤ .15, all *p*-values < 0.05).

**Conclusions:**

The German version of the BIPS appeared to be a reliable and valid measure which has the capacity to enhance future research on body image during pregnancy in German-speaking populations.

**Electronic supplementary material:**

The online version of this article (10.1186/s12884-019-2386-4) contains supplementary material, which is available to authorized users.

## Background

Body image refers to a psychological representation of the body and has been described as a multifaceted construct comprising cognitive, emotional, perceptual, and behavioral components [1]. During pregnancy, body size and shape change rapidly over a relatively short 40-week period and pregnancy-related physical symptoms become more pronounced [2]. These physical changes push women further from a socio-culturally prescribed thin-ideal [3], which, according to common sociocultural theories of body image, makes pregnancy a particular risk period for body image disturbances [4]. Body image disturbances during pregnancy have been linked to adverse health outcomes including maternal depression and low self-esteem [5, 6], restrained eating, impaired maternal-fetal attachment, obesity, reduced intention to breast feed and smoking behavior [5], which also have serious implications for the fetal development. Maternal body image during pregnancy may also be an important predictor of postpartum body image and maternal ante- and postpartum weight regulation [7–10].

It is hypothesized that pregnant women are likely to re-evaluate their body image standards in order to adapt to the rapid changes of their body [5, 11]. However, results with regard to the development of body image concerns from pre-pregnancy to pregnancy and over the course of pregnancy are mixed. Some findings indeed suggest a rather stable [12, 13] or even improved body image during pregnancy compared to pre-pregnancy [14, 15], indicating that body image standards may be relaxed during pregnancy. Pregnancy may be considered a time where weight gain is necessary and socially accepted and therefore less stigmatized than in other periods of a woman’s life [4, 16] and the reproductive role may be valued more highly than physical appearance [15, 17]. Furthermore, weight gain during pregnancy may be perceived ‘transient’ and unique to the childbearing experience which would buffer against body image disturbances [3, 15]. There is, however, also evidence that at least some pregnant women may find this re-evaluation of standards difficult, and that a substantial subgroup experiences clinically relevant body image concerns [18]. Moreover, a number of studies suggest that some women experience a significantly more negative body image during pregnancy compared to pre-pregnancy [19], and a decline of body satisfaction over the course of pregnancy with inconsistent findings with regard to particular risk periods during pregnancy [11, 19, 20].

The inconsistency of these findings may reflect the complexity of body image during the perinatal period which, to date, has not been fully understood. It may also reflect the different methods (study designs, measures of body image) applied in these studies which makes direct comparisons across studies difficult [15]. Most research on body image during pregnancy has relied on measures developed for and validated among non-pregnant populations or has focused exclusively on the degree of satisfaction with appearance, mostly measured along a single continuum [4, 5, 11]. Qualitative research, however, has emphasized that during pregnancy different body parts or features of body image become more salient (e.g. stomach, breasts, skin changes) [3] and therefore body image during pregnancy may qualitatively differ from body image in non-pregnant populations. Furthermore, the functionality as well as the public nature and sexual attractiveness of the pregnant body may be unique features of body image during pregnancy and body image experiences may be influenced by expectations about the pregnant body and perceived changes to a greater extent [21]. These body image features are not or only poorly covered by body image measures developed for non-pregnant populations [4, 21], and they also go beyond a single continuum ranging from satisfaction to dissatisfaction with body image. There is also evidence that body image measures developed for non-pregnant populations will be associated with different types of biases when used in pregnant populations. Fuller-Tyszkiewicz et al. [2], for example, found non-invariant item intercepts across pregnancy and in comparisons between pregnant and non-pregnant women for the Body Attitudes Questionnaire (BAQ) – a measure developed for and validated in non-pregnant populations but widely used to measure body image in pregnancy.

In the light of these results, Watson et al. [4] developed a self-report measure which was designed to assess body image during pregnancy (Body Image in Pregnancy Scale, BIPS) taking into account the particular aspects of body image which have been found to become more salient during pregnancy, but still being consistent with the multifaceted theoretical understanding of body image. The construction of the BIPS items was based on the themes extracted from several qualitative studies focusing on body image experiences in pregnant women [3, 21–24], but also informed by established measures of body images designed for non-pregnant populations. The BIPS was designed to cover key features of body image, including body dissatisfaction, importance and ideals of body image, pregnancy-related body changes, functioning of the pregnant body, sexual attractiveness, and appearance-related behaviors. In a study including 251 pregnant women, the BIPS demonstrated good internal consistency and retest reliability, as well as convergent and incremental validity with measures of depression and self-esteem [4]. The BIPS can therefore be a valuable tool allowing for a more comprehensive assessment of body image during pregnancy and may help to improve our understanding of the complex nature of body image during pregnancy.

In summary, more research based on measures suitable to cover the special features of body image during pregnancy is necessary to improve our knowledge about the nature, course and possible adaptation mechanisms of body image during pregnancy. Although there is evidence that body image issues are common in German-speaking populations [25] most of our knowledge on body image during pregnancy stems from studies conducted in English speaking countries and therefore more research efforts are needed on an international level. To improve comparability of studies it would be beneficial to evaluate the BIPS in internationally diverse samples. To achieve this goal in our study, we aimed to develop a German version of the BIPS and evaluate its psychometric properties. Following the procedures described for the English-language original version of the BIPS we also aimed to test its convergent and incremental validity with regard to the constructs body dissatisfaction, depression, and self-esteem. As suggested by the authors of the original BIPS [4] we extended the test of validity for the German version to the constructs anxiety and eating disorder psychopathology. These constructs were considered adequate criteria for the validity assessment as they have been shown to be related to body image concerns during pregnancy [5, 6].

## Methods

### Participants and procedures

Participants for an online study were recruited via social media posts and websites targeted at pregnant women, flyers and advertisement in gynecological and midwife practices and magazines, as well as word of mouth and snowballing techniques. Recruitment took place between April and October 2018. Women interested in participating in the study followed a link to an online survey platform where they were offered detailed information about the study, including its aims, confidentiality and inclusion criteria with the option to download study information material. A provision of informed consent was necessary for the complete online-survey to open. Informed consent was provided by agreeing to several consent questions including, for example, information on data confidentiality, on how to contact the researcher, and the voluntary nature of study participation. If interested women disagreed on any of the consent questions, they could not proceed to the online survey. Participating women could voluntarily take part in a lottery with a chance of winning one out of 10 parents’ guidebooks as incentive after completing the online survey. Inclusion criteria were a) age ≥ 18 years, b) current pregnancy ≥ 4 week’s gestation, c) sufficient German language skills to take part in the online survey, and d) informed consent.

### Measures

Participants were asked to provide demographic and pregnancy-related variables, including age, marital status, household income, education, parity, weeks’ gestation as well as body height and pre-pregnancy body weight to calculate pre-pregnancy Body Mass Index (BMI = body weight (kg)/(body height (m))^2^).

#### Body image in pregnancy scale

The English-language original version of the BIPS [4] consists of 36 items to be answered on a 5-point response scale. Scale reference points differ depending on the aspect of body image measured (e.g. body image dissatisfaction, body image importance). The items cover seven factors: ‘preoccupation with physical appearance’ (six items), ‘dissatisfaction with facial features’ (four items), ‘sexual attractiveness’ (six items), ‘prioritizing appearance over body functioning’ (five items), ‘appearance-related behavioral avoidance’ (three items), and ‘dissatisfaction with body parts’ (six items). The past week prior to the assessment was chosen as the time referent, so that the women should think of their current pregnancy and weeks’ gestation while responding the items. Mean scores (range 1–5) are calculated for every subscale with higher scores indicating greater body image disturbance.

The BIPS items were translated adhering to a stepwise forward-backward translation rationale [26]. In a first step BIPS items were translated forward from English into German language independently by two native German-speaking study members. In a second step, translations were reviewed and discussed in the study team and a preliminary German version was approved. Ambiguities were resolved with the help of the author of the original English version. Then (step 3) a native English-speaker translated this preliminary version into English. In a last fourth step, the back-translated version was reviewed and compared with the English original version by the study team and the original author. Discrepancies were resolved by discussion and a first final German version of the BIPS (BIPS-G) was approved. Cognitive interviews based on thinking aloud and retrospective verbal probing techniques using this first German version were conducted with five pregnant women (mean age: 28.60; *SD* = 0.80; mean gestational week: 29.40; *SD* = 8.66) to evaluate the comprehensibility of the translated items. Overall, the comprehensibility and relevance of the items was good. Results from the cognitive interviews were used to slightly revise formulations of a small number of items (see Additional file 1 for the final BIPS-G version; the English-language original version of the BIPS is published in Watson et al. [4]).

#### Related constructs

Body dissatisfaction was measured using the German version of the Body Shape Questionnaire (BSQ; [27], [‘Fragebogen zum Figurbewusstsein’, FFB; [28]). The FFB total score ranges from 34 to 204 with higher scores indicating higher body dissatisfaction. The reliability, factorial and convergent validity of the FFB have been shown previously [29]. The reliability of the FFB sum score in the present sample was α = .97. Depression was assessed using the German version of the Edinburgh Postnatal Depression Scale (EPDS, [27, 30]). The EPDS sum score ranges from 0 to 30 with higher scores indicating a higher severity of depressive symptomatology. The German version of the EPDS has been shown to have a good reliability [30] and the applicability of the EPDS for the use during pregnancy has been established [31]. In our sample, the reliability of the EPDS sum score was α = .88. Anxiety was measured using the German version of the General Anxiety-7 Screener (GAD-7; [32, 33]). The GAD-7 sum score ranges from 0 to 21 with higher scores indicating more severe anxiety symptom levels. There is evidence for the reliability and validity of the German version of the GAD-7 in the general population [33]. The internal consistency of the GAD-7 sum score in the present study was α = .85. Self-esteem was measured with the revised German version of the 10-item Rosenberg Self-Esteem Scale (RSE; 34). The RSE sum score ranges from 0 to 30 with higher scores indicating higher self-esteem. The reliability and validity of the German version of the RSE has been established [34, 35]. The reliability of the RSE sum score in the present sample was α = .92. Eating disorder psychopathology was measured using the German version of the Eating Disorder Examination-Questionnaire (EDE-Q; [36, 37]). Twenty-two items cover the subscales restraint, eating concern, weight concern and shape concern. Subscale means and a mean Global Score indicating the overall eating disorder psychopathology were calculated. The German version of the EDE-Q has been shown to have a good internal consistency on Global Score and subscale level [38]. The reliability in the present sample was .78 ≤ α ≤ .93 for the EDE-Q subscales. The reliability of the Global Score was α = .95.

### Statistical analyses

All statistical analyses were conducted using SPSS 25. The significance level was set to α = .05.

#### Item analyses

Standard item analyses were calculated including item mean scores and standard deviations, item difficulties (%) [p_i_ = ((x̅_i_ – min (x_i_))/(max (x_i_)-min (x_i_))*100 with x̅_i_ = mean of item i; min (x_i_) = minimal value on item i; max (x_i_) = maximum value on item i] and corrected item-total correlations. Item difficulties range from 0 to 100%. Higher item difficulties indicate a higher agreement to the items, i.e. the higher the item difficulty the higher the probability of body image disturbances on the respective item.

#### Factor structure

Due to the novelty of the BIPS and the fact that the factor structure of the English original version of the measure has only been established in one study based on exploratory factor analysis thus far [4], we chose an exploratory over a confirmatory approach to test the factor structure of the newly developed German version. Following the procedure described in Watson et al. [4] a principal axis factor analysis (PAF) with oblique rotation (PROMAX) was performed on the 36 translated BIPS items. Factors were assumed to correlate as they all measure different aspects of pregnancy body image. Items with poor factor loadings on their primary factor (< .40) or high cross-loadings (difference of less than .20) were deleted. Extraction criteria were eigenvalues > 1 (Kaiser-Guttman criterion), visual inspection of the scree-plot, and the minimum average partial test (MAP test; [39]). The MAP test was performed following the procedure described by O’Connor [40]. In a second step, a second PAF was conducted using the number of factors extracted in the first analysis.

#### Reliability

The internal consistency of the final item set was calculated using Cronbach’s α. Mean inter-item correlations were calculated to inform about subscale homogeneity.

#### Validity

Convergent validity was assessed by calculating Pearson’s correlations between BIPS subscales and theoretically related constructs, including body dissatisfaction (FFB), depression (EPDS), anxiety (GAD-7), global self-esteem (RSE), and eating disorder symptomatology (EDE-Q). Multiple hierarchical linear regression analyses were conducted to test the incremental validity of the BIPS. Therefore, depression, anxiety, self-esteem and the EDE-Q Global Score of eating disorder psychopathology were separately regressed onto the BIPS subscales (third step) after controlling for body dissatisfaction (FFB, second step) and sociodemographic characteristics (BMI, age, household income).

## Results

### Study sample

A total of 291 pregnant women gave informed consent and completed the survey. Participating women were aged between 19 and 41 years (M = 31.26, SD = 4.17). About 14% (*n* = 41) were in the first, 30% (*n* = 86) in the second and 56% (*n* = 164) in the third trimester of pregnancy (mean weeks’ gestation = 26.08, SD = 9.77; range: 5–41). Of the participating women, 99% (*n* = 289) were living with a partner, about 51% (*n* = 147) reported to already have children, and the majority (77.3%, *n* = 225) reported to have completed at least 12 years of school education. About 55% (*n* = 159) reported a net household income of 3000 euros and above which exceeds the median of about 2800 euros reported by the German Federal Office of Statistics for the German general population in 2017. The majority had German nationality (97.3%, *n* = 283). Mean pre-pregnancy BMI was at the upper end of the normal weight range (M = 24.38 kg/m^2^, SD = 4.96, range: 16.61–48.88).

### Item analyses

Item characteristics of the 36 BIPS-G items are displayed in Table 1. Item means on the five-point response scale (range 1–5) ranged from 1.25 (item 33) to 3.60 (item 18). Most items were positively skewed except for items 17–21. For most items, kurtosis was negative. Items 32 and 33 showed highest values for skewness and kurtosis and exceeded the acceptable range for factor analysis [41]. Both items were omitted in the final factor analysis. Item difficulties ranged from 6.36% (item 33) to 65.12% (item 18) with a mean item difficulty of 34.40% (SD = 12.64).

**Table 1:** Item characteristics of the BIPS German version (BIPS-G)

Item #	Range	Mean	SD	Skewness	Kurtosis	Item difficulty p_i_ (%)
1	1–5	2.41	1.23	0.45	−1.03	35.14
2	1–5	2.51	1.26	0.33	−1.20	37.71
3	1–5	2.55	1.29	0.25	−1.30	38.66
4	1–5	2.31	1.40	0.62	−1.09	32.82
5	1–5	1.66	1.04	1.48	1.09	16.58
6	1–5	2.49	1.38	0.46	−1.12	37.20
7*	1–5	2.91	1.15	0.09	−0.87	47.68
8*	1–5	2.77	1.16	0.32	−0.72	44.33
9^a^	1–5	2.35	1.32	0.54	−1.06	33.76
10*	1–5	2.37	0.99	0.65	0.06	34.19
11*	1–5	2.20	0.90	0.78	0.33	29.98
12*	1–5	2.17	0.91	0.62	0.08	29.21
13*	1–5	2.91	1.14	0.08	−0.70	47.68
14*	1–5	2.50	1.04	0.41	−0.43	37.46
15	1–5	2.77	1.28	0.15	−1.26	44.33
16	1–5	2.90	1.42	0.05	− 1.45	47.42
17	1–5	3.13	1.33	−0.22	−1.29	53.18
18	1–5	3.60	1.31	−0.68	−0.81	65.12
19	1–5	2.86	1.20	−0.13	−1.02	46.56
20	1–5	3.01	1.32	−0.09	−1.21	50.26
21	1–5	3.21	1.22	−.0.40	−0.95	55.15
22	1–5	2.34	1.37	0.64	−0.96	33.51
23	1–5	1.96	1.16	1.15	0.38	24.05
24	1–5	2.34	1.30	0.65	−0.74	33.51
25	1–5	2.03	1.16	0.84	−0.27	25.86
26^a^	1–5	2.28	1.17	0.56	−0.72	32.04
27	1–5	2.08	1.09	0.56	−0.65	26.98
28	1–5	2.10	1.21	0.75	−0.51	27.41
29	1–5	2.38	1.25	0.48	−0.91	34.45
30	1–5	1.92	1.15	0.97	−0.15	23.02
31	1–5	2.10	1.39	0.84	−0.86	27.49
32	1–5	1.30	0.83	3.28	10.62	7.47
33^a^	1–5	1.25	0.73	3.21	10.33	6.36
34^a^	1–5	1.51	0.84	1.62	2.01	12.71
35	1–5	2.17	1.28	0.74	−0.61	29.30
36	1–5	2.20	1.25	0.64	−0.76	29.98

Note. ^a^item later omitted from final analysis; *reverse-coded items; higher values indicate higher body image concerns

### Factor structure

In a first step, all 36 BIPS-G items were entered in the PAF. Both, the Kaiser-Meyer-Olkin measure (KMO = .85) and Bartlett’s Test of Sphericity (χ^2^(df = 630) = 6176.58, *p* < .001) indicated the factorability of the BIPS-G items. Although the extraction led to eight factors with eigenvalues > 1, the scree-plot and Velicer’s [39] MAP-Test supported a six-factor solution. After rotation, factor-loadings were inspected and four items with factor-loadings < .40 on the main factor were identified (items 9, 26, 32, 33). These items were omitted in the second PAF. Results of the final PAF including the 32 remaining BIPS-G items and a forced six-factor solutions are displayed in Table 2. The six factors accounted for a total of 57.2% (from first to last: 25.4, 11.3, 7.3, 5.7, 4.4, 3.2%) of the explained variance in the BIPS-G items. Inter-factor correlations ranged from .02 to .57 with seven of the 15 (46.7%) pairwise correlations exceeding .30. Our final six-factor solution differed slightly from the seven-factor solution proposed for the English language original BIPS [4]. Factors 2–6 replicated the factors found in the English original version resembling the subscales ‘dissatisfaction with strength-related aspects of the pregnant body’ (F2, items 15–21), ‘dissatisfaction with body parts’ (F3, items 27–31), ‘dissatisfaction with complexion’ (F4, items 22–25), ‘prioritization of appearance over function (F5, items 10-14), and ‘concerns about sexual attractiveness’ (F6, items 5-8). The items of the originally separate factors ‘preoccupation with physical appearance’ and ‘appearance-related behavioral avoidance’ collectively loaded on the first factor and were therefore combined on one factor named ‘preoccupation with appearance’ (F1, items 1–4, 34–36) (see Table 2 for factor loadings and communalities of the final 32-item 6-factor solution). One item from the original ‘concerns about sexual attractiveness’ factor, one item from the original ‘dissatisfaction with body parts’ factor and two items of the original ‘appearance-related behavioral avoidance’ factor have been omitted in the final BIPS German version.

**Table 2:** Factorial validity and reliability of the BIPS-G^a^

Item #	F1	F2	F3	F4	F5	F6	*h* ^2^	Corrected item-total correlation r_tt_	Cronbach’s α if item deleted
	α = .91	α = .91	α = .82	α = .85	α = .79	α = .83			
1	**.837**	−.085	−.013	.004	.020	.000	.677	.78	.89
2	**.756**	.037	.048	−.054	−.104	.038	.583	.72	.89
3	**.777**	−.014	−.026	−.008	−.021	.048	.608	.76	.89
4	**.628**	−.033	.039	.020	.024	.124	.532	.68	.90
5	.221	.008	.003	−.049	.076	**.527**	.504	.64	.80
6	.162	.018	.017	.008	.094	**.578**	.549	.66	.80
7*	−.071	−.025	.014	−.003	−.068	**.810**	.558	.64	.80
8*	007	−.022	−.024	.069	.005	**.847**	.716	.73	.75
9	–	–	–	–	–	–	–	–	–
10*	−.002	−.020	.131	.010	**.458**	.011	.242	.42	.79
11*	.143	−.139	.054	.133	**.597**	−.130	.466	.55	.75
12*	.024	−.059	.039	.003	**.825**	−.034	.689	.71	.70
13*	−.046	.073	−.105	−.104	**.685**	.037	.448	.55	.75
14*	−.068	.127	−.081	−.002	**.723**	.079	.532	.61	.73
15	.130	**.588**	.033	.035	−.045	.073	.481	.64	.90
16	−.014	**.771**	.024	−.122	−.064	.061	.629	.74	.89
17	.022	**.854**	−.069	.069	−.098	−.070	.688	.76	.89
18	.027	**.821**	−.109	.057	.019	−.103	.592	.71	.90
19	.070	**.708**	.074	.008	.067	−.019	.582	.72	.89
20	−.133	**.746**	.095	−.032	.095	.122	.651	.74	.89
21	−.072	**.838**	.019	.029	.058	−.053	.661	.76	.89
22	.038	.011	.132	**.721**	.043	−.066	.583	.69	.82
23	−.022	.012	−.099	**.794**	−.041	.104	.625	.69	.82
24	−.013	.023	.079	**.781**	−.002	−.050	.627	.74	.79
25	−.057	.006	−.118	**.799**	−.003	.052	.620	.67	.82
26	–	–	–	–	–	–	–	–	–
27	.135	.042	**.513**	.065	.007	.003	.383	.55	.80
28	−.034	−.081	**.881**	.036	.006	−.011	.713	.75	.74
29	.213	.087	**.518**	.018	−.092	.044	.474	.60	.79
30	−.176	−.028	**.814**	.023	−.034	.044	.586	.66	.77
31	.007	.083	**.658**	−.160	.074	−.064	.449	.54	.81
32	–	–	–	–	–	–	–		
33	–	–	–	–	–	–	–	–	–
34	**.767**	−.044	−.065	−.079	.045	−.061	.508	.65	.90
35	**.807**	.036	−.013	.028	.027	−.042	.651	.75	.89
36	**.846**	.101	−.035	.037	−.004	−.083	.681	.77	.89

Note. ^a^Factor loadings (pattern matrix) and communalities from the final Principal Axis Factor analysis (PAF) with oblique rotation (PROMAX with Kaiser-normalization), corrected item-total correlations, and Cronbach’s *α* if item deleted for the final BIPS-G item set (32 items); F1, preoccupation with appearance; F2, dissatisfaction with strength-related aspects of the pregnant body; F3, dissatisfaction with body parts; F4, dissatisfaction with complexion; F5, prioritization of appearance over function; F6, concerns about sexual attractiveness; BIPS-G, Body Image in Pregnancy Scale-German version; α, Cronbachs’s alpha; *h*^2^, communalities; * reverse-coded items; − items omitted from final analysis; factor loadings on the item’s primary factor are printed in bold type

Item characteristics of the final 32-item version of the BIPS-G were as follows: Item means on the five-point scale ranged from 1.51 (item 34) to 3.60 (item 18). Item difficulties ranged from 12.71% (item 34) to 65.12% (item 18) with a mean item difficulty of 36.22% (SD = 11.36) (Table 1). Corrected item-total correlations on a subscale level ranged from .65 to .78 for the ‘preoccupation with appearance’ subscale, from .64 to .76 for the ‘dissatisfaction with strength-related aspects of the pregnant body’ subscale, from .54 to .75 for the ‘dissatisfaction with body parts’ subscale, from .67 to .74 for the ‘dissatisfaction with complexion’ subscale, from .42 to .61 for the ‘prioritization of appearance over function’ subscale, and from .64 to .73 for the ‘concerns about sexual attractiveness’ subscale (Table 2).

### Reliability

Internal consistencies of the final BIPS-G 32-item version were good to excellent ranging from α = .79 for the ‘prioritization of appearance over function’ scale to α = .91 for the ‘preoccupation with appearance’ and ‘dissatisfaction with strength-related aspects of the pregnant body’ subscales. The internal consistency of the BIPS-G subscales would not have benefited from removing further items (Table 2). Mean inter-item correlations ranged from .43 (subscale ‘prioritization of appearance over function’) to .59 (subscales ‘preoccupation with appearance’, ‘dissatisfaction with strength-related aspects of the pregnant body’) indicating adequate subscale differentiability.

### Validity

Table 3 displays descriptive statistics on the subscale level for the BIPS-G and hypothetically related constructs. As expected, BIPS-G subscales mainly showed significant positive bivariate correlations with measures of body dissatisfaction, depression, anxiety, and eating disorder psychopathology. Furthermore, BIPS-G subscales were negatively correlated with self-esteem. Bivariate correlations with body dissatisfaction were strongest for the BIPS-G ‘preoccupation with appearance’ and ‘concerns about sexual attractiveness’ subscales. Depression correlated most strongly to the BIPS-G subscales ‘dissatisfaction with strength-related aspects of the pregnant body’ as did anxiety. Eating disorder psychopathology was most strongly related to the BIPS-G ‘preoccupation with appearance’ subscale. Lower self-esteem was most strongly related to the BIPS-G subscale ‘concerns about sexual attractiveness’. Twenty-nine of the 54 (53.7%) pairwise correlations were in the moderate to high range (Table 4). The hierarchical multiple linear regression analyses showed that the BIPS-G subscales significantly predicted depression (ΔR^2^ = .15, *p* < .001, step III), anxiety (ΔR^2^ = .10, *p* < .001, step III), self-esteem (ΔR^2^ = .06, *p* = .001, step III), and overall eating disorder psychopathology (ΔR^2^ = .03 *p* < .001, step III) after controlling for sociodemographic characteristics and body dissatisfaction in steps I and II indicating the incremental validity of the BIPS-G subscales. Regression coefficients are displayed in Table 5 showing that dissatisfaction with strength-related aspects of the pregnant body independently predicted depression, anxiety, and self-esteem. Concerns about sexual attractiveness independently predicted depression, and preoccupation with appearance was an independent predictor of overall eating disorder psychopathology (Table 5).

**Table 3:** Descriptive statistics for BIPS-G subscales, FFB, EPDS, GAD-7, RSE and EDE-Q

Scale	M	SD	min-max
BIPS-G preoccupation with appearance	2.24	0.99	1.00–4.86
BIPS-G dissatisfaction with strength-related aspects of the pregnant body	3.07	1.04	1.00–5.00
BIPS-G dissatisfaction with body parts	2.12	0.93	1.00–4.80
BIPS-G dissatisfaction with complexion	2.17	1.04	1.00–5.00
BIPS-G prioritization of appearance over function	2.43	0.73	1.00–4.60
BIPS-G concerns about sexual attractiveness	2.46	0.97	1.00–5.00
FFB body dissatisfaction	68.49	32.17	34.00–187.00
EPDS depression	7.25	5.78	0.00–28.00
GAD-7 anxiety	5.14	3.98	0.00–19.00
RSE self-esteem	23.48	5.80	5.00–30.00
EDE-Q restraint	0.69	1.06	0.00–5.00
EDE-Q eating concern	0.52	0.84	0.00–4.80
EDE-Q weight concern	1.05	1.32	0.00–6.00
EDE-Q shape concern	1.29	1.44	0.00–6.00
EDE-Q global score	0.89	1.01	0.00–4.42

Note. *BIPS-G* Body Image in Pregnancy Scale-German version, *FFB* Fragebogen zum Figurbewusstsein (German version of the Body Shape Questionnaire), *EPDS* Edinburgh Postnatal Depression Scale, *GAD-7* General Anxiety Disorder-7 Screener, *RSE* Rosenberg Self-Esteem Scale, *EDE-Q* Eating Disorder Examination Questionnaire

**Table 4:** Convergent validity of BIPS-G subscales^a^

	Body dissatisfaction (FFB)	Depression (EPDS)	Anxiety (GAD-7)	Self-Esteem (RSE)	Restraint (EDE-Q)	Eating Concern (EDE-Q)	Weight Concern (EDE-Q)	Shape Concern (EDE-Q)	Overall eating disorder psychopa-thology (EDE-Q global score)
BIPS-G preoccupation with appearance	.82***	.25***	.25***	−.34***	.61***	.60***	.75***	.79***	.81***
BIPS-G dissatisfaction with strength-related aspects of the pregnant body	.32***	.47***	.39***	−.35***	.04	.30***	.25***	.31***	.26***
BIPS-G dissatisfaction with body parts	.37***	.22***	.20**	−.24***	.15*	.29***	.34***	.34***	.33***
BIPS-G dissatisfaction with complexion	.19**	.12*	.16**	−15**	.13*	.12	.15*	.17**	.16**
BIPS-G prioritization of appearance over function	.37***	.04	.04	−.17**	.24***	.27***	.39***	.40***	.39***
BIPS-G concerns about sexual attractiveness	.58***	.35***	.28***	−.38***	.30***	.40***	.53***	.60***	.55***

Note. ^a^Bivariate Pearson correlations of BIPS-G subscales with measures of body dissatisfaction, depression, anxiety, self-esteem, and eating disorder psychopathology; *p < .05; **p < .01; ***p < 0.001; Bonferroni-corrected threshold: *p* < 0.001 (all correlation coefficients with *** remain significant after Bonferroni correction); *BIPS-G* Body Image in Pregnancy Scale-German version, *FFB* Fragebogen zum Figurbewusstsein (German version of the Body Shape Questionnaire), *EPDS* Edinburgh Postnatal Depression Scale, *GAD-7* General Anxiety Disorder-7 Screener, *RSE* Rosenberg Self-Esteem Scale, *EDE-Q* Eating Disorder Examination-Questionnaire

**Table 5:** Incremental valitiy of BIPS-G subscales^a^

	Depression (EPDS)				Anxiety (GAD-7)				Self-Esteem (RSE)				Overall eating disorder psychopathology (EDE-Q)			
	B	SE B	β	Δ R^2^	B	SE B	β	Δ R^2^	B	SE B	β	Δ R^2^	B	SE B	β	Δ R^2^
*Step 1 (df = 3287)*				.06***				.03*				0.4**				.07***
Constant	6.22	3.09			6.29	2.16			19.42	3.13			−.76	.54		
Age	.08	.08	.06		.00	.06	.00		−.002	.09	−.002		.03	.02	.14*	
Net household income	−.53	.13	−.25***		−.24	.09	−.16**		.44	.13	.21**		−.06	.02	−.16**	
BMI	.08	.06	.07		.02	.05	.02		.04	.06	.04		.04	.01	.19**	
*Step 2 (df = 4286)*				.10***				.08***				.22***				.70***
Constant	4.69	2.94			5.30	2.08			21.72	2.77			−1.48	.27		
Age	.04	.08	.09		−.03	.06	−.03		.07	.08	.05		.01	.01	.05	
Net household income	−0.45	.12	−.21***		−.18	.09	−.12*		.32	.12	.15**		−.02	.01	−.05	
BMI	0.02	.06	.02		−.02	.04	−.02		.12	.06	.11*		.01	.01	.05	
Body dissatisfaction (FFB)	.06	.01	.32***		.04	.01	.30***		−.09	.01	−.48***		.03	.001	.86***	
*Step 3 (df = 10, 280)*				.15***				.10***				.06**				.03***
Constant	2589	3.00			3.71	2.21			23.80	2.98			−1.60	.29		
Age	−.01	.07	−.01		−.06	.05	−.06		.10	.07	.07		.01	.01	.05	
Net household income	−.33	.12	−.15**		−.11	.09	−.07		.24	.12	.11*		−.02	.01	−.06*	
BMI	−.03	.06	−.03		−.05	.04	−.06		.15	.06	.14*		.01	.01	.05	
FFB Body dissatisfaction	.04	.02	.25**		.02	.01	.20*		−.08	.02	−.46***		.02	.002	.62***	
BIPS-G preoccupation with appearance	−.62	.53	−.11		−.09	0.39	−.02		.88	.53	.15		.28	.05	.28***	
BIPS-G dissatisfaction with strength-related aspects	1.86	.32	.34***		1.01	.23	.27***		−.97	.32	−.18**		−.03	.03	−.03	
BIPS-G dissatisfaction with body parts	−.06	.36	−.01		.06	.26	.01		−.23	.36	−.34		−.03	.03	−.03	
BIPS-G dissatisfaction with complexion	−.004	.29	−.001		.21	.21	.06		−.04	.29	−.01		−.004	.03	−.004	
BIPS-G prioritization of appearance over function	−.72	.44	−.09		−.45	.33	−.08		.08	.44	.01		.07	.04	.048	
BIPS-G concerns about sexual attractiveness	1.07	.39	.18**		.45	.29	.11		−.87	.39	−.15*		.02	.04	.021	

Note. ^a^Results from multiple linear hierarchical regression analysis with forced blockwise entry predicting depression, anxiety, self-esteem, and eating disorder psychopathology; sociodemographic characteristics (age, household net income, BMI) were entered in the first block, body dissatisfaction was entered in the second block, and BIPS-G subscales were entered in the third block.); *BIPS-G* Body Image in Pregnancy Scale-German version, *FFB* Fragebogen zum Figurbewusstsein (German version of the Body Shape Questionnaire), *EPDS* Edinburgh Postnatal Depression Scale, *GAD-7* General Anxiety Disorder-7 Screener, *RSE* Rosenberg Self-Esteem Scale, *EDE-Q* Eating Disorder Examination Questionnaire; *p < .05; **p < .01; ***p < .001

## Discussion

The BIPS is a recently developed and validated self-report measure which allows for a tailored and multifaceted assessment of body image during pregnancy by also covering body parts and body image features which become more salient or are unique during pregnancy [21]. Our study developed and examined the psychometric properties and validity of a German version of the BIPS and sought to extend validity tests to the constructs of anxiety and eating disorder psychopathology.

In the final exploratory factor analysis we identified six distinct, yet related, factors resembling ‘preoccupation with appearance’, ‘dissatisfaction with strength-related aspects of the pregnant body’, ‘dissatisfaction with body parts’, ‘dissatisfaction with complexion’, ‘prioritization of appearance over function’, and ‘concerns about sexual attractiveness’. The number of extracted factors differed slightly from the English-language original version of the BIPS for which seven factors were extracted. We also chose to omit four items (items 9, 26, 32, 33) from the final BIPS-G version due to poor factor loadings on the main factor and excess values for skewness and kurtosis (items 32 and 33). In the original BIPS items 32 and 33 contributed to the factor ‘appearance-related behavioral avoidance’ containing three items in total (items 32–34). In our analysis, the remaining item 34 asking whether women have restrained eating during pregnancy in order to feel thinner during pregnancy loaded on the first factor ‘preoccupation with appearance’. Therefore we added item 34 to the first factor and omitted the factor ‘appearance-related avoidance’ in the BIPS-G. This is plausible as restrained eating may be more a sign of high levels of preoccupation with appearance than avoidance behavior. The ‘appearance-related behavioral avoidance’ subscale showed the lowest reliability (.69) in the original BIPS [4], possibly due to the brevity of the scale. The remaining factors in our final analysis reflect exactly those found for the original BIPS and subscales showed good to excellent reliability. The BIPS-G, consistent with the results for the original English-language version [4], thus also reflects most of the themes relevant for body image experiences during pregnancy derived from qualitative studies [3, 21–24]. Body image importance and ideals are reflected in the ‘preoccupation with appearance’ factor and body changes are distributed over several factors (e.g. ‘satisfaction with strength’, ‘satisfaction with complexion’) leading to a layered structure of the BIPS which was replicated for the German version.

The BIPS-G also demonstrated convergent validity with measures of body dissatisfaction, depression, anxiety, self-esteem and eating disorder psychopathology reflecting previous findings with regard to psychological correlates of negative body image during pregnancy [5]. Findings indicated that higher body image disturbances were associated with higher levels of body dissatisfaction, depression, anxiety, eating disorder psychopathology and lower self-esteem. Correlations with body dissatisfaction were lowest for the ‘dissatisfaction with complexion’ subscale. This may reflect the fact that skin changes are indeed a specific feature of body image becoming more salient during pregnancy and are usually not covered by body image measures developed for non-pregnant populations. Consistent with the results for the English-language original version of the BIPS [4], the BIPS-G demonstrated incremental validity by improving the prediction of depression and self-esteem. In our study, the ‘satisfaction with strength’ and ‘sexual attractiveness’ scales contributed most importantly to the improvement of the prediction of depression and self-esteem over sociodemographic characteristics and body dissatisfaction. Our study additionally revealed that BIPS-G subscales – most importantly preoccupation with appearance and dissatisfaction with strength-related aspects – improved the prediction of anxiety and overall eating disorder psychopathology beyond sociodemographic characteristics and body dissatisfaction. This result underlines the importance of the novel subscales and the relevance of theses body image facets among pregnant women [21]. These results indicate that the salient features of body image during pregnancy may indeed qualitatively differ from those among non-pregnant women [4, 21] making direct comparisons between pregnant and non-pregnant populations difficult. When looking at the descriptive results for the BIPS-G we found that, on average, women scored around the scale midpoints, which was consistent with the results for the original BIPS [4]. Highest mean scores were found on the subscale ‘dissatisfaction with strength-related aspects of the pregnant body’ which was also found to be important in the prediction of depression, anxiety and self-esteem.

The results of our study have to be interpreted in the light of several limitations. Data are based on online self-report questionnaires and are cross-sectional in nature. Our sample was quite homogeneous with regard to socio-economic variables. The majority of our sample was highly educated, living with a partner, of middle to high income, and of German nationality. Our sample may therefore not be representative of the general pregnant population. It cannot be ruled out that self-selection of study participants led to an overrepresentation of women with body image concerns in our sample. Thus, further testing the psychometric properties and validity of the BIPS-G in more diverse samples is warranted. Due to the cross-sectional nature of our study, we were not able to assess trajectories of body image experiences during the perinatal period. It would be informative to evaluate the capacity of the BIPS-G to identify critical risk periods where body image disturbances are heightened and its sensitivity to change. Future research should therefore focus on prospective studies including several assessment points during the perinatal period. Due to the explorative nature of the BIPS-G factor structure in the present study, cross-validation of the suggested factor structure in other samples is necessary. It would also be interesting to evaluate the validity of the BIPS in ethnically diverse samples. Overall, the BIPS-G is a self-report measure assessing subjective body image experiences. Further testing its correspondence with different (objective) measurement methods would help to clarify its ecological validity (e.g. partner ratings, eye-tracking methods, behavioral measures).

## Conclusions

Despite these limitations we can conclude that to the best of our knowledge, the BIPS is the first measure which allows for a multifactorial and tailored assessment of body image during pregnancy and has been validated among pregnant women. It allows for a more comprehensive and accurate assessment of body image experiences specific to pregnancy and focuses on body image during pregnancy as a multifaceted construct rather than just focusing on body dissatisfaction [4]. Uçar et al. [42] recently described the Body Image Concerns during Pregnancy Scale which was also developed and psychometrically tested in a sample of pregnant women. To date, however, this scale is only available in Turkish language and lacks an evaluation of its convergent and incremental validity. Furthermore, the scale does not assess concerns about strength-related aspects of the pregnant body, which appeared to be an important feature of pregnancy body image in our study and also improved the prediction of depression, anxiety and self-esteem. In our study, the BIPS-G has demonstrated good psychometric properties and convergent and incremental validity were established. The BIPS therefore appeared to be a reliable and valuable tool for improving our understanding of the complex nature of body image during pregnancy and enhancing future research directions on body image during pregnancy in German-speaking populations.

## Additional file

Additional file 1: Body Image in Pregnancy Scale – German version (BIPS-G). (DOCX 21 kb)

## Data Availability

The datasets used for analysis during the current study are available from the corresponding author upon request.

## Abbreviations

BIPS: Body Image in Pregnancy Scale
BIPS-G: Body Image in Pregnancy Scale-German version
BSQ: Body Shape Questionnaire
EDE-Q: Eating Disorder Examination-Questionnaire
EPDS: Edinburgh Postnatal Depression Scale
FFB: Fragebogen zum Figurbewusstsein
GAD-7: General Anxiety-7 Screener
PAF: Principal axis factor analysis
RSE: Rosenberg Self-Esteem Scale
