# Supplementary material for: Measuring body image during pregnancy: psychometric properties and validity of a German translation of the Body Image in Pregnancy Scale (BIPS-G)
Source: BMC Pregnancy Childbirth. 2019 Jul 12;19:244. doi: 10.1186/s12884-019-2386-4 (PMC6626371; doi:10.1186/s12884-019-2386-4)
Supplement: Supplementary file 1 — Body Image in Pregnancy Scale – German version (BIPS-G). (DOCX 21 kb) [file 12884_2019_2386_MOESM1_ESM.docx]

**Additional file 1.** Body Image in Pregnancy Scale – German version (BIPS-G)

This file contains the German language version of the Body Image in Pregnancy Scale (BIPS-G). The original English-language version of the Body Image in Pregnancy Scale along with scoring instructions is published in Watson B, Fuller-Tyszkiewicz M, Broadbent J, Skouteris H. Development and validation of a tailored measure of body image for pregnant women. Psychol Assess. 2017;29:1363–75. doi:10.1037/pas0000441 (Appendix B). Item content and numbers of the German version of the BIPS correspond to the original English-language version. Scoring instructions for the BIPS-G, which differ slightly from those of the original BIPS are given below.

Nachfolgend finden Sie eine Liste mit Aussagen zu verschiedenen Aspekten Ihres Körpers während der Schwangerschaft. Bitte beziehen Sie sich bei Ihrer Antwort auf Ihre aktuelle Schwangerschaft und dabei auf die aktuelle Schwangerschaftswoche.

Bitte wählen Sie bei jeder der folgenden Aussagen aus, inwiefern Sie dieser zustimmen.

| **In der aktuellen Schwangerschaftswoche…** | **stimme überhaupt nicht zu** | **stimme eher nicht zu** | **weder noch** | **stimme eher zu** | **stimme voll und ganz zu** |
| --- | --- | --- | --- | --- | --- |
| 1. verbringe ich viel Zeit damit, über mein Gewicht nachzudenken. (F1) | **o** | **o** | **o** | **o** | **o** |
| 2. verbringe ich viel Zeit damit, über meinen Körperumfang nachzudenken. (F1) | **o** | **o** | **o** | **o** | **o** |
| 3. verbringe ich viel Zeit damit, über meine Figur nachzudenken. (F1) | **o** | **o** | **o** | **o** | **o** |
| 4. wünsche ich mir sehr, dünner zu sein. (F1) | **o** | **o** | **o** | **o** | **o** |
| 5. ziehe ich es vor, dass mein/e Partner/in meinen schwangeren Körper nicht nackt sieht. (F6) | **o** | **o** | **o** | **o** | **o** |
| 6. ziehe ich es vor, dass andere meinen schwangeren Körper nicht nackt sehen. (F6) | **o** | **o** | **o** | **o** | **o** |
| 7. mag und schätze ich meinen schwangeren Körper in sexueller Hinsicht.* (F6) | **o** | **o** | **o** | **o** | **o** |
| 8. finde ich meinen schwangeren Körper attraktiv.* (F6) | **o** | **o** | **o** | **o** | **o** |
| *9. mache ich mir Sorgen, dass mein/e Partner/in meinen schwangeren Körper unattraktiv finden könnte.* | ***o*** | ***o*** | ***o*** | ***o*** | ***o*** |
| 10. denke ich mehr darüber nach, wie ich mich in meinem Körper fühle als darüber, wie er aussieht.* (F5) | **o** | **o** | **o** | **o** | **o** |
| 11. bin ich stärker damit beschäftigt, wie ich mich mit meinem Körper bewegen kann als damit, wie er aussieht.* (F5) | **o** | **o** | **o** | **o** | **o** |
| 12. bin ich stärker mit der Funktionsweise meines Körpers beschäftigt als damit, wie er aussieht.* (F5) | **o** | **o** | **o** | **o** | **o** |
| 13. bin ich stärker mit der Funktion meiner Brüste beschäftigt als damit, wie sie aussehen.* (F5) | **o** | **o** | **o** | **o** | **o** |
| 14. bin ich stärker mit der Funktion meines Bauches beschäftigt als damit, wie er aussieht.* (F5) | **o** | **o** | **o** | **o** | **o** |

Nachfolgend finden Sie eine Liste mit Fragen zu Ihrem Erleben rund um körperliche Veränderungen während der Schwangerschaft. Bitte beziehen Sie sich bei der Antwort auf Ihre aktuelle Schwangerschaft und dabei auf die aktuelle Schwangerschaftswoche.

Bitte wählen Sie bei den nachfolgenden Fragen aus, wie zufrieden Sie mit den jeweiligen Veränderungen sind. Sollte eine Frage für Sie während Ihrer Schwangerschaft nicht von Bedeutung sein, dann wählen Sie die Option „trifft nicht zu“ aus.

|  | **sehr zufrie-den** | **etwas zufrie-den** | **weder noch** | **etwas unzu-frieden** | **sehr unzu-frieden** | **trifft nicht zu** |
| --- | --- | --- | --- | --- | --- | --- |
| 15. Wie zufrieden sind Sie in der aktuellen Schwangerschaftswoche mit Ihrer Körperspannung? (F2) | **o** | **o** | **o** | **o** | **o** | **o** |
| 16. Wie zufrieden sind Sie in der aktuellen Schwangerschaftswoche mit Ihrer Beweglichkeit? (F2) | **o** | **o** | **o** | **o** | **o** | **o** |
| 17. Wie zufrieden sind Sie in der aktuellen Schwangerschaftswoche mit Ihrer körperlichen Kraft? (F2) | **o** | **o** | **o** | **o** | **o** | **o** |
| 18. Wie zufrieden sind Sie in der aktuellen Schwangerschaftswoche mit Ihrem Energieniveau? (F2) | **o** | **o** | **o** | **o** | **o** | **o** |
| 19. Wie zufrieden sind Sie mit den Veränderungen Ihrer Körperspannung im Verlauf der Schwangerschaft? (F2) | **o** | **o** | **o** | **o** | **o** | **o** |
| 20. Wie zufrieden sind Sie mit den Veränderungen Ihrer körperlichen Beweglichkeit im Verlauf der Schwangerschaft? (F2) | **o** | **o** | **o** | **o** | **o** | **o** |
| 21. Wie zufrieden sind Sie mit den Veränderungen Ihrer körperlichen Kraft im Verlauf der Schwangerschaft? (F2) | **o** | **o** | **o** | **o** | **o** | **o** |
| 22. Wie zufrieden sind Sie in der aktuellen Schwangerschaftswoche mit Ihrem Hautbild? (F4) | **o** | **o** | **o** | **o** | **o** | **o** |
| 23. Wie zufrieden sind Sie in der aktuellen Schwangerschaftswoche mit Ihren Haaren? (F4) | **o** | **o** | **o** | **o** | **o** | **o** |
| 24. Wie zufrieden sind Sie mit den schwangerschaftsbedingten Veränderungen Ihres Hautbilds im Verlauf der Schwangerschaft? (F4) | **o** | **o** | **o** | **o** | **o** | **o** |
| 25. Wie zufrieden sind Sie mit den Veränderungen Ihrer Haare im Verlauf der Schwangerschaft? (F4) | **o** | **o** | **o** | **o** | **o** | **o** |
| *26. Wie zufrieden sind Sie in der aktuellen Schwangerschaftswoche mit Ihren Brüsten?* | ***o*** | ***o*** | ***o*** | ***o*** | ***o*** | ***o*** |
| 27. Wie zufrieden sind Sie in der aktuellen Schwangerschaftswoche mit der Größe/Breite Ihrer Schultern? (F3) | **o** | **o** | **o** | **o** | **o** | **o** |
| 28. Wie zufrieden sind Sie in der aktuellen Schwangerschaftswoche mit Ihren Knöcheln? (F3) | **o** | **o** | **o** | **o** | **o** | **o** |
| 29. Wie zufrieden sind Sie in der aktuellen Schwangerschaftswoche mit Ihren Armen? (F3) | **o** | **o** | **o** | **o** | **o** | **o** |
| 30. Wie zufrieden sind Sie n der aktuellen Schwangerschaftswoche mit Ihren Haaren? (F3) | **o** | **o** | **o** | **o** | **o** | **o** |
| 31. Wie zufrieden sind Sie in der aktuellen Schwangerschaftswoche mit den Wassereinlagerungen in Ihrem Körper? (F3) | **o** | **o** | **o** | **o** | **o** | **o** |

Nachfolgend finden Sie eine Liste mit Fragen zu Ihren Verhaltensweisen während der Schwangerschaft. Bitte beziehen Sie sich bei Ihrer Antwort auf Ihre aktuelle Schwangerschaft und dabei auf die aktuelle Schwangerschaftswoche.

Bitte wählen Sie bei jeder der folgenden Fragen aus, wie häufig Sie sich so verhalten haben.

|  | **niemals** | **kaum** | **manch-mal** | **oft** | **ständig** |
| --- | --- | --- | --- | --- | --- |
| *32. Haben Sie es in der aktuellen Schwangerschaftswoche vermieden Sport zu treiben, weil etwas an Ihrem Körper schwabbeln könnte?* | ***o*** | ***o*** | ***o*** | ***o*** | ***o*** |
| *33. Sind Sie in der aktuellen Schwangerschaftswoche nicht zu geselligen Anlässen (z.B. Partys) gegangen, weil Sie sich wegen Ihrer Figur unwohl gefühlt haben?* | ***o*** | ***o*** | ***o*** | ***o*** | ***o*** |
| 34. Haben Sie sich in der aktuellen Schwangerschaftswoche beim Essen eingeschränkt, um sich dünner zu fühlen? (F1) | **o** | **o** | **o** | **o** | **o** |
| 35. Haben Sie in der aktuellen Schwangerschaftswoche aus Sorge um Ihre Figur das Gefühl gehabt, dass Sie Sport treiben/sich mehr bewegen sollten? (F1) | **o** | **o** | **o** | **o** | **o** |
| 36. Haben Sie in der aktuellen Schwangerschaftswoche aus Sorge um Ihr Gewicht das Gefühl gehabt, dass Sie Sport treiben/sich mehr bewegen sollten? (F1) | **o** | **o** | **o** | **o** | **o** |

Subskalen:

F1, Aussehensbezogene Sorgen; F2, Unzufriedenheit mit der Körperkraft; F3, Unzufriedenheit mit Körperteilen/-regionen; F4, Unzufriedenheit mit der Haut; F5, Priorisierung des Aussehens gegenüber der Funktionalität, F6, Sorgen bezüglich der sexuellen Attraktivität.

Subscales:

F1, preoccupation with appearance; F2, dissatisfaction with strength-related aspects of the pregnant body; F3, dissatisfaction with body parts; F4, dissatisfaction with complexion; F5, prioritization of appearance over function; F6, concerns about sexual attractiveness.

Auswertung:

* Items vor Subskalenbildung invertieren

Items in kursiver grauer Schrift: aus Skalenbildung ausgeschlossen, finale Version der BIPS-G hat 32 Items

Subskalenmittelwerte aus allen Items einer Subskala bilden; Range: 1-5; höhere Werte weisen auf starker ausgeprägtes negativen Körperbild hin; die Berechnung eines Gesamtwertes ist nicht empfohlen

Scoring instructions:

*Items to reverse code

Items in italic font are omitted from subscale scoring in the German version. The final BIPS-G contains 32 items.

All items of a subscale should be averaged so that subscale scores range from 1-5; higher subscale scores reflect greater body image disturbance. It is not recommended to calculate a total score.
